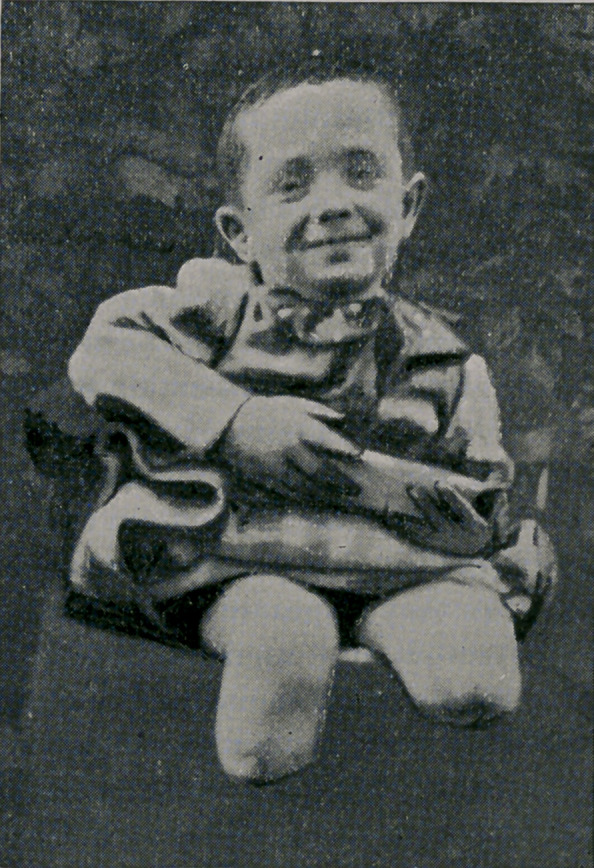# Double Synchronous Amputation of Both Legs in an Infant—Recovery

**Published:** 1893-09

**Authors:** Gregory Doyle

**Affiliations:** Syracuse, N. Y.


### DOUBLE SYNCHRONOUS AMPUTATION OF BOTH LEGS IN AN INFANT—RECOVERY.

By GREGORY DOYLE, M. D., Syracuse, N. Y.

On the 8th day of April last, Ambrose Mullin, the infant son of Michael Mullin, of 1016 Willis avenue, Syracuse, N. Y., was run over by an electric car, and suffered what was thought at the time fatal injuries. Both legs were crushed off just below the knee, and his head was so severely injured as to produce cerebral concussion, large, dark tumors being produced on the forehead and occiput. He suffered also severe contusions over the sternum. About an hour after the accident I reached the little patient, and found him in a complete stupor and very anemic from the immense loss of blood. By hypodermic stimulation he rallied sufficiently to warrant me in removing the mangled members. The amputations were made carefully, but rapidly, as I knew celerity to be a strong element in possible success. During the operation hypodermic stimulation was persistently kept up, and we had the pleasure of seeing our almost hopeless patient slowly but steadily rally from the shock. He made a good recovery, without any noteworthy incident. The stumps healed rapidly, with good cushioned ends, so that, hereafter, artificial limbs can be worn with comfort, and the child may grow up to be a useful and honorable citizen. At present he is able to go about the house and yard on his knees, and is healthy and cheerful, as the above photograph indicates.

The child was born January 10, 1891 ; two legs were amputated April 8, 1893 ; his age at the time of the operation was, therefore, a little over two years. As far as I can learn, this is the youngest child on record that has recovered from a synchronous amputation of both legs.

I was ably assisted in this unusual operation by Drs. J. W. Knapp, N. L. Mulvey, Joseph Roth, and Gregory Reidy.

## Figures and Tables

**Figure f1:**